# Estimate of the Biological Dose in Hadrontherapy Using GATE

**DOI:** 10.3390/cancers14071667

**Published:** 2022-03-25

**Authors:** Yasmine Ali, Caterina Monini, Etienne Russeil, Jean Michel Létang, Etienne Testa, Lydia Maigne, Michael Beuve

**Affiliations:** 1 Institut de Physique des 2 Infinis de Lyon, Université Claude Bernard Lyon 1, CNRS/IN2P3, 4 rue Enrico Fermi, 69622 Villeurbanne, France; ali@ip2i.in2p3.fr (Y.A.); monini@ip2i.in2p3.fr (C.M.); e.testa@ip2i.in2p3.fr (E.T.); michael.beuve@univ-lyon1.fr (M.B.); 2 Laboratoire de Physique de Clermont, Université Clermont Auvergne, CNRS/IN2P3, 4 Avenue Blaise Pascal, 63178 Aubière, France; russeil@clermont.in2p3.fr; 3 CREATIS, Université Claude Bernard Lyon 1, CNRS UMR5220, Inserm U1294, INSA-Lyon, Université Lyon 1, 69373 Lyon, France; jean.letang@insa-lyon.fr

**Keywords:** biological dose, Monte Carlo, Geant4-DNA, LPCHEM, GATE, mMKM, NanOx

## Abstract

**Simple Summary:**

This study presents the implementation of a biological dose module using the Monte Carlo software, GATE. Both mMKM and NanOx biophysics models of cell survival predictions were used as input. The code was validated in terms of biological dose, relative biological effectiveness and cell survival against experimental data from the HIMBC (Hyogo, Japan) ion beam line.

**Abstract:**

For the evaluation of the biological effects, Monte Carlo toolkits were used to provide an RBE-weighted dose using databases of survival fraction coefficients predicted through biophysical models. Biophysics models, such as the mMKM and NanOx models, have previously been developed to estimate a biological dose. Using the mMKM model, we calculated the saturation corrected dose mean specific energy $z_{1D}^{*}$ (Gy) and the dose at 10% *D*_10_ for human salivary gland (HSG) cells using Monte Carlo Track Structure codes LPCHEM and Geant4-DNA, and compared these with data from the literature for monoenergetic ions. These two models were used to create databases of survival fraction coefficients for several ion types (hydrogen, carbon, helium and oxygen) and for energies ranging from 0.1 to 400 MeV/n. We calculated *α* values as a function of LET with the mMKM and the NanOx models, and compared these with the literature. In order to estimate the biological dose for SOBPs, these databases were used with a Monte Carlo toolkit. We considered GATE, an open-source software based on the GEANT4 Monte Carlo toolkit. We implemented a tool, the BioDoseActor, in GATE, using the mMKM and NanOx databases of cell survival predictions as input, to estimate, at a voxel scale, biological outcomes when treating a patient. We modeled the HIBMC 320 MeV/u carbon-ion beam line. We then tested the BioDoseActor for the estimation of biological dose, the relative biological effectiveness (RBE) and the cell survival fraction for the irradiation of the HSG cell line. We then tested the implementation for the prediction of cell survival fraction, RBE and biological dose for the HIBMC 320 MeV/u carbon-ion beamline. For the cell survival fraction, we obtained satisfying results. Concerning the prediction of the biological dose, a 10% relative difference between mMKM and NanOx was reported.

## 1. Introduction

Treatment Planning Systems (TPS) are software with fast calculation performances. They have been developed to maintain their performances while improving the accuracy of their analytical algorithms for dosimetry planning. However, there are still limits, especially when using ions, to correctly assess the range of particles in complex geometries with density variations [1]. Monte Carlo codes can overcome such limits. Despite being more time consuming than analytical algorithms, they are more accurate for planning doses in radiation therapy. Such codes consider tumor heterogeneity by modeling specific material properties, electron density, mass density, ionization potential, etc. [2]. Consequently, Monte Carlo toolkits have been used for medical applications. In hadrontherapy, some of these toolkits are used to provide an RBE-weighted dose (dose x relative biological effectiveness weighting factor) using databases of survival fraction coefficients predicted through biophysical models [3,4]. For example, the Monte Carlo code FLUKA [5] has been coupled with the LEM biophysical model [6] and has been adopted in the Heidelberg Ion-Beam Therapy Center (HIT) in Germany and in the National Center for Oncological Hadrontherapy (CNAO) in Italy to support both dose and RBE-weighted dose calculations performed by the analytical TPS. FLUKA has also been coupled with the mMKM model [7]. Among the existing Monte Carlo toolkits for medical applications, GATE is an open-source toolkit based on the GEANT4 Monte Carlo code [8,9,10]. The platform has been validated for clinical use in the field of light ion beam therapy using pencil beaming scanning (PBS) technique and it is currently used in different clinical centers as the independent tool for dose calculation such as in the Proton Beam Therapy Center at the Christie NHS Foundation Trust (Manchester, UK) and in the MedAustron Ion Therapy Center (Wiener Neustadt, Austria) [11,12,13]. The next step in the development of the platform for hadrontherapy applications is to estimate the biological quantities (cell survival fractions, biological dose and RBE) for hadrontherapy treatments. Therefore, in this paper, we consider the implementation of a new actor (a tool enabling the collection of information during the simulation, such as physical dose in voxels or in segmented geometry, deposit energy, etc.) called BioDoseActor, to calculate the biological dose, at a voxel scale, based on the biophysical models mMKM and NanOx when treating a patient with ion beams, typically proton and carbon ions. The microdosimetric kinetic model developed by Hawkins [14] was based on the theory of dual radiation action (TDRA), and was then adapted into the modified microdosimetric kinetic model (mMKM) by NIRS Japanese researchers [15]. In the mMKM model, the surviving fraction of cells can be predicted from the specific energy deposited into a micrometric scaled volume, called domain. The NanOx model [16] was developed to overcome the potential shortcomings of the existing models, in particular, by taking into account the impact of energy deposition at micrometric and nanometric scales, with full modeling of radiation stochastic effects. For that purpose, the NanOx model defines two types of damage that can impact the survival of cells. First, the local lethal events consisting of biological events taking place at nanometric scale leading to cell death through severe DNA damage. Second, the non-local events consisting of, for instance, the accumulation of sub-lethal DNA damage at micrometric scale and represented by the production of chemical reactive species that induce cell oxidative stress. Each model requires Monte Carlo Track Structure Code (MCTS) calculations to define specific energy or chemical species produced in a cell nucleus. In this work, we considered two MCTS codes: LPCHEM [17] and Geant4-DNA [18,19,20]. LPCHEM is used in the NanOx model and Geant4-DNA is the only open-source MCTS code available at this time that has been developed to calculate direct and indirect damage to molecules and cells. Both codes are able to perform the simulation of ionizing radiation consequences (physical, physico-chemical and chemical stages) to water. In a previous paper, those codes were detailed and benchmarked; we showed that they can provide good results for the simulation of specific energy spectra at micrometric and nanometric scales and time-dependent G values necessary for NanOx and mMKM models [21].

In radiation biology experiments, cell survival rate as a function of absorbed dose can be fitted with the linear quadratic (LQ) model [22,23] as proposed in Equation (1), where *α* and *β* parameters describe the cell’s radiosensitivity, and *D* is the dose. Biophysical models were developed to provide predictive values for those parameters. The *α* value reflects cell death from lethal damage caused by a single incident particle. Therefore, this parameter is highly dependent on the linear energy transfer (LET).

(1) $$S=e^{-\alpha D-\beta D^{2}}$$

First, in order to tackle the impact of MCTS code on microdosimetry quantities, we compared the saturation corrected dose mean specific energy $z_{1D}^{*}$ (Gy) and the dose at 10% of survival $D_{10}$ described in the mMKM algorithm using LPCHEM and Geant4-DNA MCTS codes. Then, we estimated the *α* values as a function of the LET for human salivary gland (HSG) cell line with mMKM and NanOx models. HSG cell survival has been intensively used to validate the mMKM model at different beam qualities [24]. Our results were compared with the literature each time it was possible. Then, we described the mathematical formalism of the BioDoseActor; this new actor used, as input data, pre-calculated *α* and *β* parameters produced with mMKM and NanOx models for monoenergetic ions (hydrogen, carbon, helium and oxygen) and for energies ranging from 0.1 to 400 MeV/n. Finally, we estimated cell survival fractions, biological doses and RBE for a 320 MeV/n carbon-ion clinical beam from Hyogo Ion Beam Medical Center (HIBMC) in Japan, and compared them with biological experiments performed by Kagawa et al. [25].

## 2. Materials and Methods

### 2.1. Cell Survival Predictions Using mMKM and NanOx Models for Monoenergetic Ions

We focused our study on the human tumor cells from salivary glands (HSG) cell line and its response to hydrogen, helium, carbon and oxygen ion mono-energetic beams (from 0.1 MeV/n to 400 MeV/n). Experimental *α* values were only available for helium and carbon mono-energetic beams and were taken from the PIDE (Particle Irradiation Data Ensemble) project [26], and other values came from mMKM calculations from the literature. Errors associated with the experimental measurements have not been reported. Hereafter, we detail the parameters used in the NanOx and mMKM models. For the mMKM model, we calculated $z_{1D}^{*}$ and $D_{10}$ for HSG cell line using LPCHEM and Geant4-DNA MCTS codes and compared them with data from Inaniwa et al. [24].

#### 2.1.1. NanOx Parameters for HSG Cell Line

A detailed description of the NanOx model has been provided by Cunha et al. [27]. In this work, we do not detail the model framework but focus only on the descriptions of the parameters required to simulate the cell survival coefficients. The NanOx model input parameters can be classified into two categories.

First, in order to estimate the cell survival due to local lethal events, an effective local lethal function *F* was calculated (Equation (2)). *F* follows a monotonical increase with specific energy z deposited in local targets (10 nm) uniformly distributed within the cell nucleus with radius *R_sv_* (μm). The outcome of the construction procedure was close to an error-like function as described in the work of Monini et al. [28,29]. It consisted of deriving coefficients related to local lethal events from the representative data (experimental *α* values) in order to constrain *F* and optimize its parameters. A threshold value $z_{0}$, a factor *σ* controlling the width of the increase, and a function maximum *h*, were used.

(2) $$F\left(z\right)=\frac{h}{2}\;\left[1+\mathrm{erf}\left(\frac{z-z_{0}}{\sigma }\right)\right]$$

Second, the contribution of global events was derived from the two following parameters: the coefficient *β_G_* (Gy) obtained from the *β* coefficient for a reference radiation, and the sensitive volume associated with global events (corresponding to the one associated with local lethal events in the present version of NanOx). We report, in Table 1, the different NanOx input parameters that were estimated for the HSG cell line using the LPCHEM MCTS [29].

The actual outcome of NanOx is the mean cell survival averaged over all irradiation configurations. Cell survival can then be calculated as a function of the dose for a monoenergetic beam. The resulting curve can be fitted with a linear-quadratic model in order to obtain *α* and *β* parameters.

#### 2.1.2. mMKM Parameters for HSG Cell Line

A detailed description of the mMKM has been provided by Kase et al. [15] and Inaniwa et al. [24], therefore in this work we will not detail the model framework and only focus on the description of the parameters required to simulate the cell survival coefficients.

For *α* values predicted by the mMKM model, we retrieved predictions from Chen et al. [30] and Russo et al. [31] that used different sets of parameters and codes. We decided to use LPCHEM and Geant4-DNA track structure codes and followed the methodology of Magro et al. [32] using the set of input parameters defined by Inaniwa et al. [24]. The mMKM parameters were: cylindrical domain radius *R_d_* (μm), the nucleus radius *R_n_* (μm), the constant *α*_0_ (Gy) that represents the initial slope of the survival fraction curve at the limit value of LET = 0, and the reference survival coefficient *β* that is a constant term. The reference set of parameters have been reported in Table 2 for the HSG cell line [24].

As these input parameters have been determined using the radial dose provided by the Kiefer–Chatterjee model with the work of the Japanese researchers developing the mMKM [15], it is important to verify these parameters for the LPCHEM and Geant4-DNA codes. Indeed, unlike the Kiefer–Chatterjee model, LPCHEM and Geant4-DNA are MCTS codes that do not rely on the radial dose estimation but on a stochastic calculation of the specific energy. First, we calculated the saturation corrected dose mean specific energy $z_{1D}^{*}$ ((Gy) (Equation (3)) using LPCHEM and Geant4-DNA, and compared these distributions with Inaniwa et al. [24].

(3) $$\begin{array}{c} z_{1D}^{*}=\frac{z_{0}^{2}\int_{0}^{\infty }\left(1-e^{-{\left(\frac{z}{z_{0}}\right)}^{2}}\right)f_{1}\left(z\right)dz}{\int_{0}^{\infty }zf_{1}\left(z\right)dz} \end{array}$$

where $f_{1}\left(z\right)$ is the probability density of the specific energy *z* deposited by a single energy-deposition event in the domain, and $z_{0}$ is the saturation-corrected specific energy (Equation (4)).

(4) $$\begin{array}{c} z_{0}=\frac{{\left(R_{n}/R_{d}\right)}^{2}}{\sqrt{\beta \left(1+{\left(R_{n}/R_{d}\right)}^{2}\right)}} \end{array}$$

Then, using the $z_{1D}^{*}$ values calculated with LPCHEM and Geant4-DNA, we estimated α and the dose at 10% of survival ($D_{10}$) for HSG cells as a function of the LET (see Equations (5) and (6)). We finally compared our results with the work of Inaniwa et al. and validated these $D_{10}$ values using experimental data from Furusawa et al. [34].

(5) $$\alpha =\alpha_{0}+\beta z_{1D}^{*}$$

(6) $$\begin{array}{c} D_{10}^{\;}=\frac{-\alpha +\sqrt{\left(\;\alpha^{2}-4\beta \ln0.1\right)}}{2\beta } \end{array}$$

### 2.2. Prediction of Biological Dose, RBE and Cell Survivals for Spread out Bragg Peaks (SOBP)

The BioDoseActor aims to calculate biological quantities at the voxel scale in CT-scan based geometry within the GATE Monte Carlo simulation. The actor is attached to the voxelized volume of interest, taking into account the matrix resolution and position within the coordinate system. Each voxel of the matrix is indexed, and recovers energy deposited by incoming ions and nuclear fragments. Cell survival fractions $S_{mix}\left(D\right)$ are predicted as a function of the dose *D*, using the parametrization of the linear quadratic (LQ) model:

(7) $$\begin{array}{c} S_{mix}\left(D\right)=e^{-\left(\;\alpha_{mix}\;D+\beta_{mix\;}^{}D^{2}\right)} \end{array}$$

(8) $$\begin{array}{c} \alpha_{mix}=\underset{t}{\sum }\underset{i}{\sum }f_{t,i}\;\alpha_{t,i} \end{array}$$

(9) $$\begin{array}{c} \sqrt{\beta_{mix}}=\underset{t}{\sum }\underset{i}{\sum }f_{t,i}\sqrt{\beta_{t,i}} \end{array}$$

where $\alpha_{mix}$ and $\sqrt{\beta_{mix}}$, respectively, are the mean values of $\alpha_{t,i}$ and $\sqrt{\beta_{t,i}}$ weighted by the deposited dose fraction *f*, and where α and *β* are the coefficients associated with the ion type *t* and kinetic energy *i* (approximation proposed by Kanai et al. [35]).

(10) $$\begin{array}{c} f_{t,i}=\frac{Edep_{t,i}}{Edep_{}} \end{array}$$

When α and *β* coefficients are not available in the data base for a given kinetic energy, a linear interpolation is performed.

This “Kanai approximation” has been tested and adopted by the Japanese researchers at the NIRS (National Institute of Radiobiological Sciences, Chiba, Japan) who obtained satisfactory results in 1999 [36]. The approach has since been adopted by the GSI (German Heavy Ion Research Center, Darmstadt, Germany) in 2000 [6], and also the HIT (Heidelberg Ion-Beam Center, Heidelberg, Germany).

Biological dose and RBE were then deduced from calculated survival fractions. The biological dose $D_{bio}$ was obtained using Equation (11), with *α_ref_* and *β_ref_*, the coefficients estimated with a reference X-ray beam.

(11) $$\begin{array}{c} D_{bio}=\frac{-\alpha_{ref}+\sqrt{\alpha_{ref}^{2}+4\;\beta_{ref\;}^{}\left(\alpha_{mix}D+\beta_{mix\;}^{}D^{2}\right)}}{2\;\beta_{ref}^{}} \end{array}$$

With the estimation of the biological dose, we estimated the RBE (Equation (12)), the ratio between the biological dose $D_{bio}$ and the physical dose *D*.

(12) $$\begin{array}{c} RBE=\frac{D_{bio}}{D} \end{array}$$

### 2.3. BioDoseActor Algorithm

**Figure 1:**
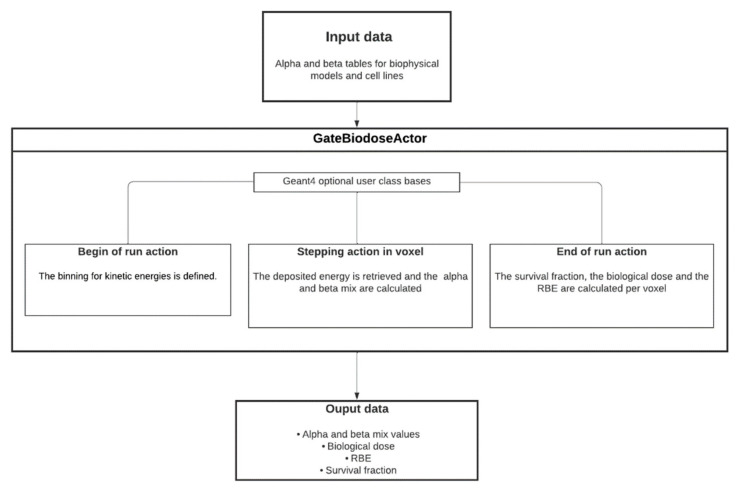
Algorithm of the BioDoseActor.

Figure 1 shows a diagram describing the algorithm of the BioDoseActor. Here, the input files (ASCII files) were the databases of survival fraction coefficients *α* and *β*, for HSG cell line, calculated with the chosen biophysical models mMKM and NanOx. In the future, databases for other cell lines of interest will be implemented. Table A1 and Table A2 gather databases from mMKM and NanOx models for the HSG cell line.

The BioDoseActor algorithm is based on a new numerical implementation of the Kanai equations (Equations (6) and (7)). The methodology relies on the linear interpolation of the *α* and $\sqrt{\beta }$ coefficients. The chosen energy ranges are set to ensure the validity of this approximation. The BioDoseActor considers that all energy losses by ions are deposited along their trajectories without considering secondary electrons (secondary electrons with ranges lower than the cut value are not tracked and the energy is assigned to the ion energy deposition).

More precisely, for each step of an ion of type *T* and energy *E*, the deposited energy is used to update (Equations (6)–(8)) the coefficients $\alpha_{mix}$ and $\sqrt{\beta_{mix}}$ of the voxel where the step takes place. The BioDoseActor algorithm is represented in Figure 1. Particular care should be taken with the step length, which should not be too long, to avoid having an aliased estimate, nor too small, to avoid a prohibitive calculation time.

In this study we approximated the computation of the biological dose, assuming that the water volume corresponded to a homogeneous HSG tissue. As output, an ASCII file was listed for each voxel of the irradiated volume, the index, the (x, y, z) coordinates, the $\alpha_{mix}$ value (Gy^−1^), the $\beta_{mix}$ value (Gy^−2^), the physical dose (Gy), the biological dose (Gy) and the RBE value. However, as the voxel size can be millimetric or sub millimetric, the BioDoseActor uses C++ maps to store and exploit data. Maps are associative containers that store elements formed by a combination of a key value and a mapped value. Maps provide several advantages over objects such as lists, arrays and vectors, as they are internally represented as a binary search tree. Therefore, data insertion, deletion and access are fast and proportional to log(*n*) where *n* is the number of elements in the map.

### 2.4. HIBMC 320 MeV/u Carbon Ion Beam Line

Heavy ion medical accelerators in Chiba (HIMAC) and in Hyogo (HIBMC) have been used to irradiate different cell lines in order to estimate the biological parameters (alpha and beta values). We decided to model a simplified version of those beam lines. The geometrical setup has been reproduced according to the literature [37]. The geometry has been validated by comparing the dose deposition with the dose reported in the literature. In the work of Kagawa et al. [25], the survival fraction and the biological dose were measured for an HSG cell line irradiated with a 320 MeV/n SOBP in HIBMC clinical beam center. We reproduced the experience by irradiating a phantom of water with a dose of 2.4 Gy at the isocenter of the SOBP.

In order to reproduce the experimental measurements settings, we modeled the 320 MeV/n carbon ion beam source with a radius of 7.5 cm, as in the work of Kagawa et al. [25], with a field irradiation size of 15 cm × 15 cm. The irradiated HSG cells were irradiated attached to a flask wall and encompassed in a 7 cm × 15 cm irradiation field. We chose to model the phantom as a box with a section of 15 cm × 15 cm. The deepest position of the pristine peak in the phantom was 220 mm. The phantom was then split along the z-axis in 1 mm slices to obtain 250 slices in total. We chose the QGSP_BIC_HP physics list as recommended in the field of hadrontherapy. Regarding secondaries production, we applied relatively high cut values (1 m) to prevent any secondary electron generation. The SOBP was produced with a ridge filter made of aluminum (light material in order to reduce ion scattering). Its design was intended to provide a uniform biological dose over the SOBP, i.e., a constant survival fraction of HSG cells, in our case. As no information has been detailed in the literature about ridge filter characteristics, we performed a non-negative least squares regression using Python in order to determine the closest parameters to be able to reproduce a 6 cm width SOBP. This SOBP was reproduced with the range shifter thicknesses and beam weights as described in Table 3.

## 3. Results

### 3.1. Cell Survival Predictions Using mMKM and NanOx Models

#### 3.1.1. $z_{1D}^{*}$ and *D*_10_ Values

**Figure 2:**
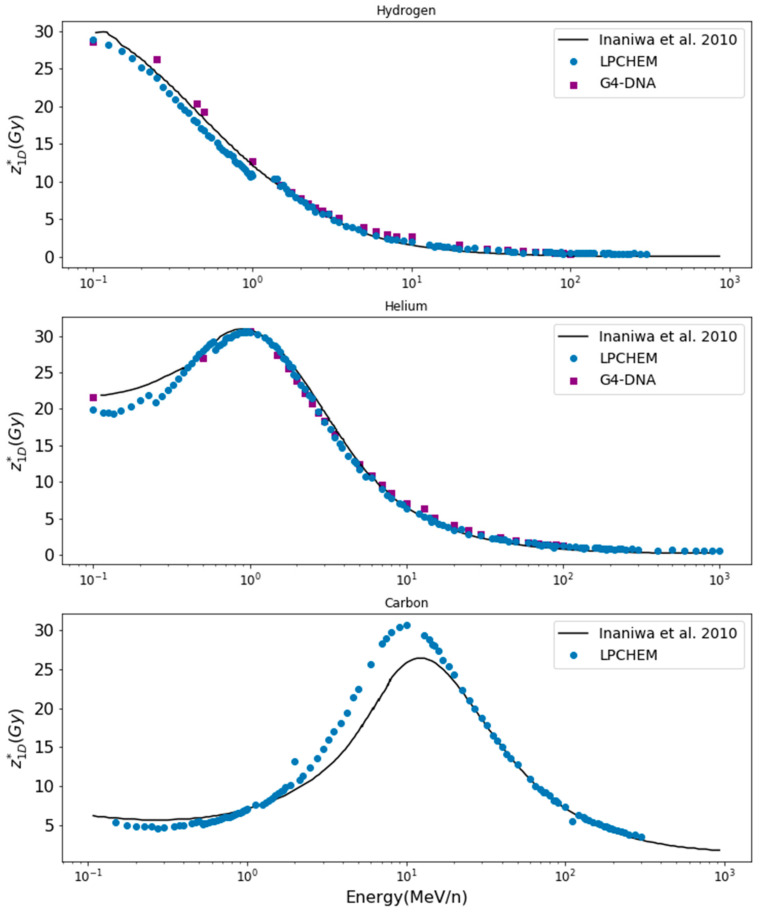
$z_{1D}^{*}$ values as a function of the kinetic energy of hydrogen, helium and carbon ions for HSG cells. Values from Inaniwa et al. were obtained from the track structure of the Kiefer–Chatterjee model [24].

**Figure 3:**
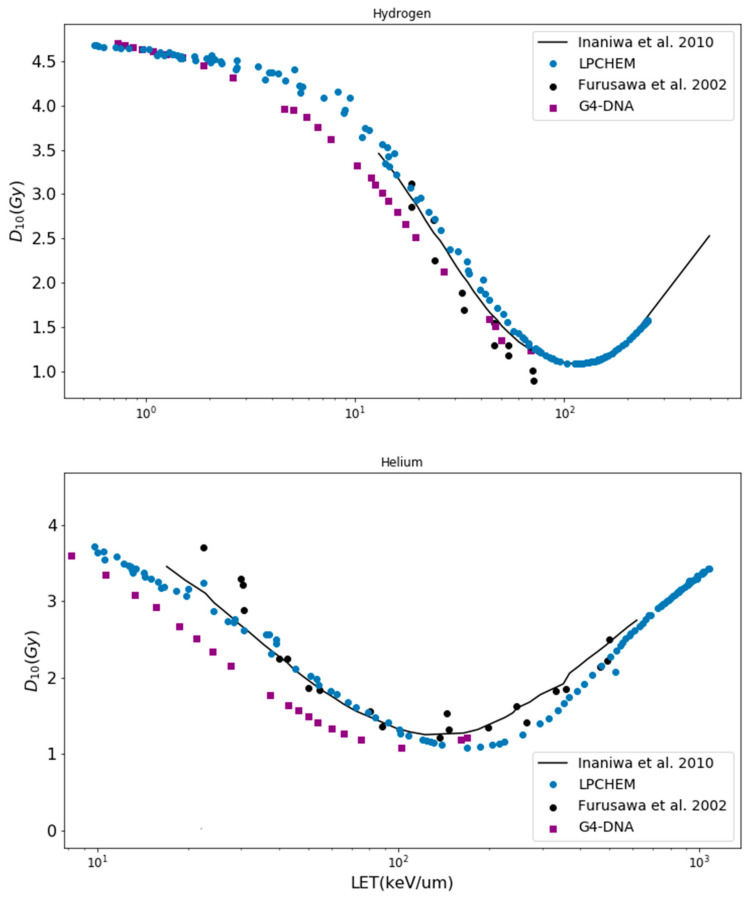
*D*_10_ values under aerobic conditions as a function of LET for helium and carbon beams for HSG cells. Geant4-DNA and LPCHEM were compared with the values from Inaniwa et al. [24] (using the track of the Kiefer–Chatterjee model) and the experimental data from Furusawa et al. [34].

Figure 2 shows the comparison of $z_{1D}^{*}$ values for hydrogen, helium and carbon ions. Hydrogen, helium, carbon and oxygen ions for kinetic energies up to 400 MeV/n were simulated with LPCHEM, but only hydrogen and helium ions up to 100 MeV/n were simulated with Geant4-DNA (upper range limit for the ionization process). Figure 3 shows the comparison of the dose at 10% of survival (*D*_10_) calculated with LPCHEM and Geant4-DNA for hydrogen and helium ions as a function of LET for HSG cell line, in addition to the values obtained by Inaniwa et al. [24] (using the track structure of the Kiefer–Chatterjee model) and Furusawa et al. [34] (experimental data).

#### 3.1.2. Predictions of *α* Values as a Function of LET

**Figure 4:**
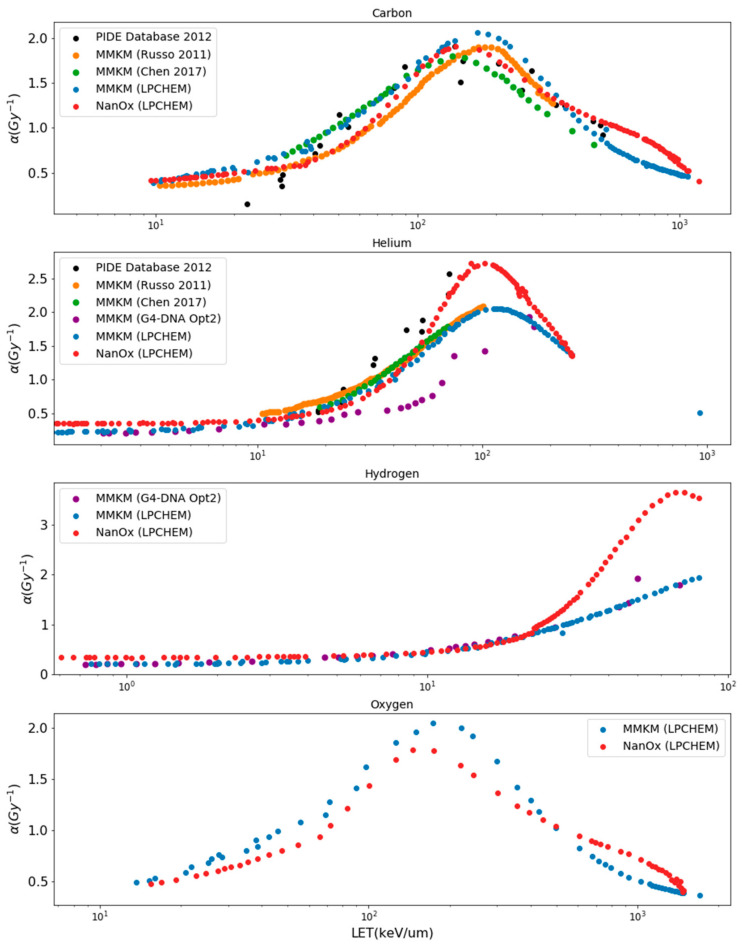
Predictions of *α* values as a function of LET for the HSG cell line in response to irradiations with carbon, helium, hydrogen, and oxygen monoenergetic ions, for mMKM and NanOx models, using LPCHEM and Geant4-DNA MCTS codes. For carbon and helium ions, our results were compared to Chen et al. [30], Russo et al. [31] and to the PIDE database [26].

Figure 4 shows predictions of *α* values with LET increasing for the HSG cell line for hydrogen, helium, carbon and oxygen ions. The predicted *α* and *β* values are reported in Appendix A and Appendix B for the NanOx and mMKM models.

- Concerning carbon ions, *α* values reproduced the PIDE experimental data trend for all authors;
- Concerning helium ions, *α* values calculated with the NanOx model were in close agreement with the PIDE experimental data. mMKM predictions from Russo et al. [31] and Chen et al. [33] resulted in close predictions except between 50 and 70 keV/μm. Higher discrepancies were observed between Geant4-DNA and PIDE experimental data;
- Concerning hydrogen ions, there were no experimental data nor predictions available in the literature. mMKM predictions, calculated with either LPCHEM or Geant4-DNA, and the NanOx model predictions, led to close results up to 25 keV/μm. For higher LET values, the NanOx model resulted in higher values than mMKM;
- Concerning oxygen ions, there were no experimental data nor predictions available in the literature. NanOx and mMKM models using LPCHEM resulted in close *α* values.

### 3.2. Cell Survival Fractions, Biological Doses and RBE for HIBMC 320 MeV/u Carbon-Ion Beam Line

**Figure 5:**
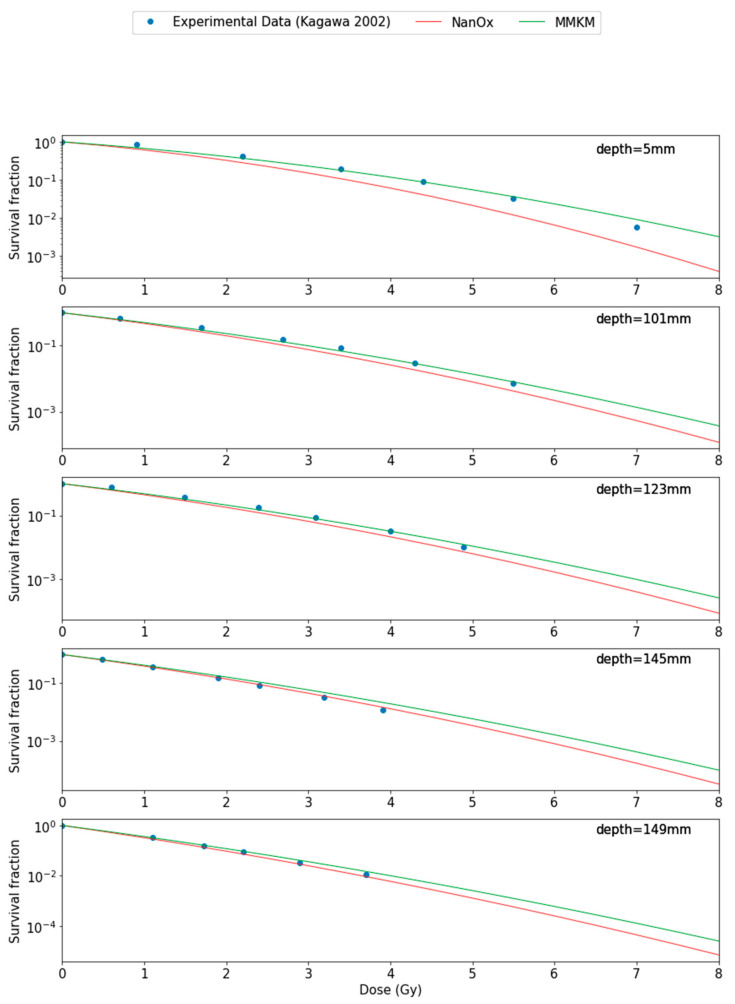
Survival fractions of HSG cells as a function of the dose using the BioDoseActor with the NanOx model (red curve) and the mMKM model (green curve) and experimental data from Kagawa et al. [25] for five positions in the SOBP: 5 mm, 101 mm, 123 mm, 145 mm, and 149 mm of the HIBMC 320 MeV/u carbon-ion beam line.

Figure 5 shows comparisons of survival fractions as a function of the dose obtained with the BioDoseActor using either the NanOx or mMKM models and experimental data from Kagawa et al. [25] for five positions in the SOBP: 5 mm, 101 mm, 123 mm, 145 mm, and 149 mm of the HIBMC 320 MeV/u carbon-ion beam line.

**Figure 6:**
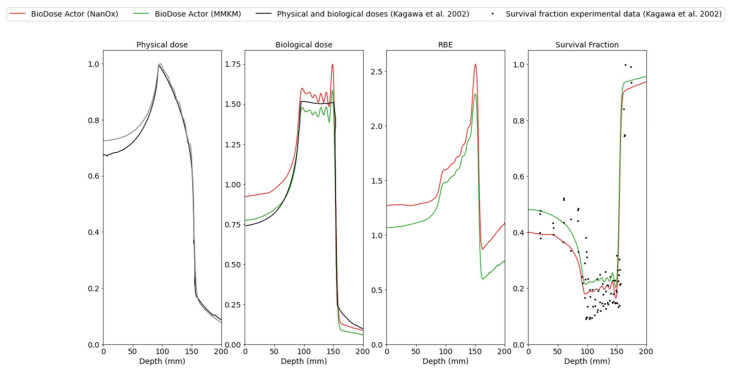
Physical dose (light grey), biological dose, RBE and survival fractions provided by the BioDoseActor as a function of target depth: NanOx model (red curve), mMKM model (green curve) and experimental data from Kagawa et al. [25] (black curves and dots) for the HIBMC 320 MeV/u carbon-ion beam line.

Figure 6 shows comparisons between the BioDoseActor outputs (physical dose, biological dose, RBE and survival fraction) and experimental data from Kagawa et al. [25] for five positions in the SOBP: 5 mm, 101 mm, 123 mm, 145 mm, and 149 mm of the HIBMC 320 MeV/u carbon beam line. For this stage, a computing time of 8 h was conducted on an Intel Xeon CPU E5-2623 v4 (4 cores, 10,240 KB Cache, 2.60 GHz, Santa Clara, CA, USA).

Relative differences (%) between biological doses obtained with NanOx and MMKM models at 5, 101, 123, 145 and 149 mm depth were, respectively, 16.3, 6.7, 6.5, 7.9, and 8.6% (see Table 4).

Table 5 shows the relative differences (%) between RBE10 and RBE50 when calculated with NanOx and MMKM, compared with Kagawa et al. For RBE10 and RBE50, the NanOx model was in better agreement with Kagawa et al. data.

## 4. Discussion

### 4.1. Validation of the mMKM Input Parameters for HSG Cell Line

One important goal of this paper was to evaluate the impact of the physical input on the predictions by the microdosimetric models. Indeed, the physical input values of the mMKM were obtained by the calculation of the specific energy through a radial dose model, namely, the Kiefer–Chatterjee model. The radial dose is defined as the average dose deposited by a single ion as a function of the distance to its trajectory. As the radial dose is an averaged quantity, the stochastic nature of the radiations is neglected [38]. Therefore, we recalculated $z_{1D}^{*}$ and *D*_10_ values by estimating the specific energy through two MCTS codes (LPCHEM and Geant4-DNA) keeping fixed the set of mMKM parameters determined with a radial dose model by Inaniwa et al.

Despite a few disparities, the values obtained with the two MCTS codes and the radial dose (Kiefer–Chatterjee) model presented similar trends. This result was coherent with the conclusions of Beuve et al. [38] and Cunha et al. [28,39], showing that the impact of specific energy fluctuations becomes relatively small for targets larger than a few micrometers, while they are dramatic at nanoscale. The fact that the predictions of the two MCTS codes were close is not surprising, according to a previous study [21]. We benchmarked the two codes for the simulation of the specific energy distributions in micrometric and nanometric targets and we concluded that for specific energy spectra in sensitive volumes at such scale, the two types of codes were in very good agreement despite a few disparities due to different cross sections.

To our knowledge, the present work is the first study of the impact of track calculations on microdosimetric quantities in the mMKM model. It would be interesting to study, in addition, the impact of various radial dose models (also known as track structure models) on the mMKM model; such a study was performed by Elsässer et al. [40] for the LEM. We could also build radial dose models from the two MCTS codes used in this study. This would allow us to not only estimate the impact of changing radial dose models on biological outcomes, but also the impact of using radial dose itself with respect to full Monte Carlo calculations. Nevertheless, the MCTS codes offer many perspectives. Indeed, whether by themselves or coupled with other simulation tools, they can produce physico-chemical and biochemical quantities such as DNA damage (PARTRAC and Geant4-DNA) or chromosomal aberrations [41,42]. Moreover, they may also integrate cellular biomolecules such as anti-oxidant [43] and oxygen concentrations [44].

Note that $z_{1D}^{*}$ values estimated with LPCHEM display discontinuities at 1 MeV/n for hydrogen ions, 0.2 and 0.6 MeV/n for helium ions and 0.4 MeV/n and 1 MeV/n for carbon ions. These discontinuities are expected, as the code does not model the charge exchanges [17] unlike Geant4-DNA, and the effective charge is chosen for each kinetic energy with a precision of 0.1. As a perspective, the effective charge could be tuned with much more precision (for instance, at 0.01) in order to make $z_{1D}^{*}$ discontinuities negligible. The generally good agreement between $z_{1D}^{*}$ and *D*_10_ values obtained with LPCHEM and Geant4-DNA and Inaniwa et al. validates the use of LPCHEM and Geant4-DNA for the production of input data for mMKM using the set of parameters of Inaniwa et al. Improvements of MCTS codes could still improve the reliability of the calculations.

### 4.2. Comparison of α Values Estimated with NanOx and mMKM Using LPCHEM and Geant4-DNA

NanOx and mMKM predicted relatively close *α* values at low and intermediate LET values (<400 keV/µm for oxygen ions, <200 keV/µm for carbon ions, <40 keV/µm for helium ions and <20 keV/µm for hydrogen ions), while, for higher LET values, *α* values predicted with NanOx were always higher. Similarities at low and intermediate LET and differences for high LET values may be explained by the different model postulates but also by the different experimental data used to constrain the model parameters: the PIDE database for NanOx and the experimental data provided in Furusawa et al. [34].

Concerning the model postulates, the two models estimate biological outcomes from specific energy in sensitive volumes. The first major difference between the two models is the fact that NanOx considers energy depositions in both nano and micro volumes, while mMKM only considers energy deposition at the micro scale. Moreover, NanOx takes into account the stochastic nature of the radiation (with full Monte Carlo simulations) unlike mMKM, that uses radial doses to estimate dose deposition around ion trajectories.

Finally, NanOx also performs Monte Carlo simulation of track number and positions allowing a direct prediction. Instead, mMKM does not explicitly simulate ion impacts and needs a saturation correction to reproduce the over-killing (substitution of *z*_1*D*_ by $z_{1D}^{*}$).

These three major differences lead NanOx to give more importance to the stochastic ion track properties (especially, the very large specific energies in the track core that can reach values of the order of 10^5^ Gy for high LET ions) and therefore to predict larger *α* values.

Finally, this study has estimated the impact of track calculations on *α* predictions by mMKM for hydrogen and helium ions using Geant4-DNA and LPCHEM (instead of the radial dose). The fact that *α* predictions using Geant4-DNA and LPCHEM were almost superimposed was expected, according to Ali et al. [21], who showed a very good agreement between specific energy spectra obtained with Geant4-DNA and LPCHEM. The deviations observed in *α* predictions for helium ions suggest further investigations on the physical models for this ion.

### 4.3. Estimate of Cell Survival Fractions, Biological Doses and RBE for Carbon and Helium Beam Lines

As a simplified version of the HIMBC line had been implemented, we observed disparities between the physical dose calculated with GATE and the physical dose retrieved from the literature (Figure 6), notably, a shift at the entrance of the SOBP with a maximum relative error of 8%. The shift was also observable in the biological dose predicted by the NanOx and mMKM models at the entrance of the SOBP: the models both overestimated the biological dose due to the overestimation of the physical dose in this area with a maximum relative error of 20% for NanOx and 10% for mMKM (Figure 6). This was consistent with the cell survival curve (Figure 5) showing a slight overestimation of cell killing by NanOx. This deviation could be corrected by fitting the $\beta_{ref}$ parameter on the cell survival curve to low LET carbon ions, as occurred for the mMKM model. Indeed $\beta_{ref}$ corresponds to the beta value for low LET radiations. It has been obtained from an average of various beta values for photons, but the beta values suffer from large fluctuations leading to large uncertainties.

However, there was a good agreement between the physical dose calculated with GATE and the reference dose in the plateau of the SOBP where the maximum relative error was 2% (Figure 6). In the plateau of the SOBP, the NanOx model overestimated the biological dose with a relative error of 6% (Figure 6). This overestimation of the biological dose therefore led to an underestimation of survival fraction compared with the experimental data values for different positions in the plateau of the SOBP (Figure 6). The mMKM model underestimated the biological dose with a relative error of 5% and led to higher predicted values of survival fraction.

While a statistical test would be useful to provide a more quantitative conclusion and a comparison between predictions and experimental data, it would require, to be meaningful, a proper estimation of both experimental and theoretical uncertainties. Regarding experimental uncertainties data, uncertainties are missing for the biological dose profile and the cell survival curves. Concerning the simulations, it is straightforward to estimate statistical fluctuations but a proper estimation of theoretical uncertainties would unfortunately require intractable calculations. The available uncertainties are summarized in Table 4 and Table 5.

## 5. Conclusions

For the first time, this work estimated the influence of specific energy fluctuations calculated by Monte Carlo simulations on mMKM predictions. Surprisingly, these resulting predictions were close to those obtained with radial dose models. Nevertheless, improving Monte Carlo simulations would allow for fixing the parameters of the biophysical models independent of the origin of the physical input. Moreover, MCTS codes, eventually coupled to other simulation tools, already offer interesting perspectives to produce chemical and biochemical quantities to be directly incorporated as input for biophysical models of cell survival.

This paper also showed the predictions of the first implementation of the BioDoseActor in GATE for an application of NanOx and mMKM models. This preliminary validation needs to be extended to other cell lines in order to propose treatment plans using mMKM or NanOx models on PBS clinical beams using patient CT scans. For the moment, the tuning of biophysical model parameters is performed from cell survival measurement of two-dimensional cell cultures. In the future, it will be important to evolve toward more complex systems as spheroid, organoid and xenografted tumors take into consideration models including immune systems and tumor dynamics.

## Figures and Tables

**Table 1:** NanOx input parameters for the HSG cell line using the LPCHEM MCTS.

$z_{0\;}\;(\mathrm{Gy})$	*σ* (Gy)	*h*	*β_G_* (Gy^−2^)	*R_SV_* (μm)
15,654	549	179,439	0.096	7

**Table 2:** mMKM input parameters for the HSG cell line from various works.

References	*R_d_* (μm)	*R_n_* (μm)	*α*_0_ (Gy^−1^)	*β_G_* (Gy^−2^)
This work, Inaniwa et al., 2010 [24], Chen et al., 2017 [33]	0.32	3.9	0.172	0.0615
Russo et al., 2011 [31]	0.20	4.6	0.313	0.0615

**Table 3:** Range shifter thicknesses (mm) and pristine peak weights for the simulation of the HIBMC SOBP 320 MeV/n carbon-ion beam line using GATE.

Range Shifter Thickness (mm)	6	7	10	11	12	16	19	20	21	24	26	28	30	32
Beam weights	1	0.82	0.12	0.14	0.65	0.66	0.10	0.24	0.29	0.35	0.24	0.23	0.020	0.35

**Table 4:** Comparison of biological doses obtained with NanOx and MMKM models at 5, 101, 123, 145 and 149 mm depth.

Depth (mm)	5	101	123	145	149
Biological dose NanOx (STD = 3.2%)	0.92	1.58	1.53	1.52	1.74
Biological dose mMKM (STD = 3.2%)	0.77	1.47	1.43	1.40	1.59
Relative difference (%)	16.3	6.7	6.5	7.9	8.6

**Table 5:** RBE10 and RBE50 obtained with NanOx, MMKM models compared with Kagawa et al. at 5, 101, 123, 145 and 149 mm depth.

Depth (mm)	5	101	123	145	149
RBE10 (Kagawa et al.)	1.23 ± 0.088	1.68 ± 0.249	1.76 ± 0.108	2.30 ± 0.113	2.56 ± 0.244
RBE10 (NanOx) (STD = 0.1%)	1.22	1.37	1.44	1.74	1.99
Relative difference (%)	0.81	18.5	18.2	24.3	22.3
RBE10 (mMKM) (STD = 0.1%)	1.01	1.31	1.39	1.67	1.83
Relative difference (%)	17.9	22.0	21	27.4	28.5
RBE50 (Kagawa et al.)	1.21 ± 0.148	1.98 ± 0.282	2.03 ± 0.232	2.91 ± 0.303	3.46 ± 0.467
RBE50 (NanOx) (STD = 0.1%)	1.26	1.59	1.70	2.17	2.54
Relative difference (%)	11.6	19.7	16.3	25.4	26.6
RBE50 (mMKM) (STD = 0.1%)	1.07	1.49	1.61	2.03	2.27
Relative difference (%)	11.6	24.7	20.6	30.2	34.3

## Data Availability

The data presented in this study are available in this article, or the from the corresponding author on reasonable request.

## Appendix A

**Table A1:** Predictions of *α* and *β* Values for the HSG Cell Line in Response to Hydrogen, Helium, Carbon, Oxygen and Oxygen Ions for the NanOx Model.

**HYDROGEN**					
**E (MeV/n)**	***α* (Gy^−1^)**	***β* (Gy^−2^)**	**E (MeV/n)**	***α* (Gy^−1^)**	***β* (Gy^−2^)**
0.1	3.52785	0.0586794	4.25	0.436973	0.0635334
0.125	3.58379	0.0219491	4.25	0.425273	0.0644498
0.15	3.64192	0.0977552	5	0.420139	0.0625937
0.175	3.64134	0.045627	6	0.406463	0.0625907
0.2	3.59205	0.0522845	7	0.389055	0.0639294
0.225	3.48742	0.0763334	7.5	0.390886	0.0518132
0.25	3.38711	0.0486352	8	0.377757	0.0648603
0.275	3.23556	0.0140717	9	0.380409	0.0416042
0.3	3.10038	0.0564686	10	0.375543	0.0629273
0.325	2.92819	0.0736448	13	0.36375	0.0637803
0.35	2.74536	0.0440922	14	0.355133	0.0663663
0.375	2.64766	0.068873	14.5	0.363962	0.039987
0.4	2.50822	0.0519665	15	0.36261	0.0653386
0.425	2.35826	0.0598118	16	0.36199	0.0194867
0.45	2.24049	0.0523686	17	0.358723	0.0634626
0.475	2.11282	0.0327819	18.5	0.354093	0.0679557
0.5	2.00902	0.0619912	20	0.361473	0.0655274
0.525	1.90411	0.048899	22.5	0.349285	0.0663717
0.55	1.81201	0.03943	25	0.348734	0.0648933
0.6	1.64405	0.0482093	30	0.340981	0.0686984
0.625	1.5553	0.0315918	35	0.338952	0.0647853
0.65	1.50294	0.0514883	40	0.33787	0.0675714
0.675	1.42643	0.0530718	42.5	0.336445	0.0712075
0.7	1.37338	0.0520192	45	0.344386	0.0727018
0.725	1.30831	0.0516787	50	0.344973	0.0997508
0.75	1.27779	0.0520332	60	0.341337	0.0779679
0.775	1.22202	0.0610424	70	0.330335	0.0788438
0.8	1.17571	0.0540186	72.5	0.339633	0.083321
0.825	1.14052	0.0496843	75	0.341254	0.0831872
0.85	1.10919	0.0522072	80	0.343223	0.088238
0.875	1.07663	0.0538031	85	0.347429	0.0845721
0.9	1.04062	0.0541082	87.5	0.340158	0.0884021
0.925	1.0123	0.0436477	90	0.343359	0.089741
0.95	0.987964	0.0546012	100	0.3399	0.0930667
0.975	0.954701	0.052234	110	0.344508	0.0972525
0.9875	0.956628	0.0588447	115	0.341763	0.010713
1	0.931771	0.058776	120	0.33716	0.093344
1.25	0.922846	0.0596302	125	0.348853	0.096485
1.375	0.829386	0.057546	130	0.348952	0.0958178
1.4375	0.811162	0.0635889	132.5	0.342216	0.108084
1.5	0.77844	0.0576283	135	0.339428	0.109774
1.5625	0.743542	0.0617835	140	0.337172	0.0978914
1.625	0.724542	0.0590762	160	0.345544	0.101325
1.6875	0.705097	0.0629384	165	0.341937	0.0939792
1.75	0.690471	0.0610498	170	0.351141	0.0839463
1.875	0.657967	0.0689717	175	0.346595	0.0956681
2	0.630247	0.0609551	180	0.331431	0.100256
2.125	0.59985	0.0558018	190	0.334884	0.104186
2.25	0.576881	0.0605689	200	0.338724	0.105441
2.375	0.560703	0.0620355	212.5	0.333464	0.094867
2.5	0.548153	0.0558827	225	0.33002	0.129717
2.75	0.521374	0.0553158	237.5	0.342825	0.0705913
3	0.49762	0.0574269	250	0.346535	0.109787
3.25	0.487405	0.0658839	275	0.339406	0.0998982
3.5	0.467097	0.0576851	300	0.339219	0.108748
3.875	0.459079	0.0697264			
**HELIUM**					
**E (MeV/n)**	***α* (Gy^−1^)**	***β* (Gy^−2^)**	**E (MeV/n)**	***α* (Gy^−1^)**	***β* (Gy^−2^)**
0.1	1.35471	0.0207273	4.625	0.913462	0.074134
0.115	1.34943	0.068132	4.8	0.894276	0.0399684
0.125	1.36143	0.0561437	5	0.845962	0.0545597
0.135	1.36702	0.0479669	5.5	0.788932	0.0548308
0.15	1.40449	0.0523761	6	0.729322	0.0589305
0.175	1.46176	0.0186978	7	0.65191	0.0584992
0.2	1.52903	0.0422706	7.5	0.618191	0.0546201
0.225	1.54966	0.0685532	8	0.594936	0.0590312
0.25	1.55482	0.0423817	9	0.552776	0.0556349
0.275	1.62378	0.0624645	9.5	0.537816	0.0270089
0.3	1.69296	0.0560868	10	0.521669	0.0646037
0.325	1.75868	0.0827488	12	0.492294	0.0631951
0.35	1.82571	0.09662	13	0.471319	0.0600745
0.375	1.89151	0.0417015	14	0.457	0.0540569
0.4	1.95804	0.0406013	14.5	0.454425	0.0647896
0.425	2.01788	0.0406869	15	0.447441	0.0639881
0.45	2.07856	0.0427398	16	0.442108	0.0585427
0.475	2.13803	0.0509513	17	0.427663	0.054866
0.5	2.19514	0.0360138	18.5	0.416323	0.0629227
0.525	2.24906	0.0560736	20	0.408267	0.0635374
0.55	2.30165	0.0719733	22.5	0.405099	0.0675533
0.56	2.31162	0.0589844	25	0.390136	0.0661453
0.575	2.35233	0.0283324	30	0.377674	0.0652145
0.58	2.3359	0.0610067	35	0.375329	0.0698207
0.6	2.24179	0.0365905	37.5	0.376945	0.068874
0.625	2.27969	0.048764	40	0.370289	0.0662104
0.65	2.32832	0.0571975	42.5	0.372768	0.0683548
0.675	2.36932	0.0582657	45	0.364365	0.0703231
0.7	2.40823	0.0107764	50	0.364275	0.0681571
0.725	2.44624	0.0803496	60	0.355372	0.0747071
0.75	2.47837	0.0209884	65	0.355306	0.0760348
0.775	2.51364	0.0847122	70	0.357653	0.0778917
0.8	2.54292	0.0519947	72.5	0.351378	0.0813151
0.825	2.57208	0.0609218	75	0.354749	0.0843505
0.85	2.59087	0.0639041	80	0.356145	0.0820511
0.875	2.61384	0.0825088	85	0.351307	0.087815
0.9	2.64201	0.0602317	87.5	0.359332	0.0900896
0.925	2.65902	0.0399342	90	0.353549	0.0857066
0.95	2.67573	0.0100122	100	0.352662	0.0869735
0.975	2.68649	0.0900614	110	0.350016	0.0897816
1	2.69945	0.0460438	120	0.352662	0.0924707
1.125	2.72759	0.0649546	130	0.349242	0.0993062
1.25	2.68623	0.0592119	132.5	0.350188	0.0977779
1.3125	2.67842	0.0437095	135	0.351315	0.101791
1.375	2.72147	0.0511202	140	0.351914	0.0996269
1.4375	2.6023	0.0481748	160	0.352059	0.103381
1.5	2.55898	0.0135152	165	0.348055	0.108882
1.5625	2.51015	0.0251847	170	0.349539	0.104125
1.625	2.51876	0.0820981	175	0.349366	0.097993
1.6875	2.40387	0.0796317	180	0.350419	0.104505
1.75	2.349	0.0442119	185	0.346769	0.107044
1.8	2.30672	0.0467401	190	0.342395	0.0732676
1.875	2.28272	0.0412346	195	0.349724	0.100912
1.9	2.23023	0.0968624	200	0.346889	0.116881
2	2.12334	0.0421089	212.5	0.348807	0.116918
2.125	2.05126	0.0369635	225	0.347231	0.0997025
2.25	1.91357	0.0728045	237.5	0.353986	0.110258
2.375	1.82345	0.0653356	250	0.34613	0.112309
2.5	1.73184	0.0416488	275	0.34762	0.107218
2.75	1.562	0.0705918	300	0.347978	0.101295
3	1.43353	0.0454924	400	0.344634	0.102051
3.25	1.31698	0.0449153	500	0.34295	0.109641
3.5	1.21521	0.0409911	600	0.345637	0.10687
3.75	1.13259	0.0515363	700	0.342868	0.115623
3.875	1.09196	0.0562185	800	0.341284	0.0974119
4.25	0.99491	0.0515848	900	0.3453	0.103232
			1000	0.340597	0.103002
**CARBON**					
**E (MeV/n)**	***α* (Gy^−1^)**	***β* (Gy^−2^)**	**E (MeV/n)**	***α* (Gy^−1^)**	***β* (Gy^−2^)**
0.1	0.554922	0.0174572	4.625	1.37202	0.0533761
0.15	0.507327	0.0501012	5	1.41833	0.0934593
0.175	0.496762	0.0185161	6	1.53289	0.0576861
0.2	0.498712	0.0128507	7	1.6441	0.0352824
0.225	0.507064	0.0398217	7.5	1.6905	0.0484951
0.25	0.517163	0.0321235	8	1.73733	0.0206558
0.275	0.524741	0.028797	9	1.81111	0.0270153
0.3	0.527277	0.024745	10	1.86519	0.0145637
0.35	0.562364	0.0325134	13	1.9013	0.0735242
0.375	0.581553	0.04897	14	1.88	0.0524471
0.4	0.600128	0.022651	14.5	1.86598	0.0753743
0.45	0.63878	0.0493113	15	1.84803	0.0247954
0.475	0.658849	0.0709208	16	1.80727	0.0386726
0.5	0.678314	0.0311766	17	1.75589	0.127889
0.525	0.651331	0.0378454	18.5	1.6866	0.0289437
0.53	0.654257	0.0387549	20	1.59615	0.0269112
0.55	0.668569	0.0323383	22.5	1.46482	0.02547
0.575	0.685794	0.0958369	25	1.3439	0.0377534
0.6	0.698747	0.0925473	27	1.25757	0.0699588
0.625	0.71445	0.0123321	30	1.14384	0.063285
0.65	0.729095	0.018834	32	1.08349	0.0503975
0.675	0.743807	0.033274	35	0.995382	0.0563748
0.7	0.757473	0.032298	37.5	0.937827	0.0468086
0.725	0.771899	0.029902	40	0.883756	0.0536156
0.75	0.7715	0.0258204	42.5	0.843352	0.06574
0.775	0.780193	0.0222819	45	0.806053	0.060595
0.8	0.792595	0.0115817	50	0.742661	0.0559711
0.825	0.804823	0.0254497	60	0.656602	0.0738968
0.85	0.818199	0.0192019	65	0.622154	0.0275371
0.875	0.828681	0.019282	70	0.587718	0.0599057
0.9	0.838734	0.0572171	72.5	0.581034	0.0643803
0.925	0.847032	0.0501417	75	0.579367	0.0263508
0.95	0.857266	0.0462406	80	0.570936	0.0643563
0.975	0.868805	0.0614917	85	0.557118	0.0420902
0.9875	0.875099	0.0422347	87.5	0.55305	0.064244
1	0.875006	0.08374	90	0.549996	0.060972
1.125	0.918784	0.0602353	100	0.529454	0.044553
1.25	0.920182	0.0925326	110	0.514361	0.0641633
1.3125	0.937178	0.0795297	120	0.500774	0.0656887
1.375	0.949605	0.0347631	130	0.490693	0.0572786
1.4375	0.964492	0.0309748	132.5	0.486477	0.0687478
1.5	0.981289	0.0190316	135	0.484113	0.0336333
1.5625	0.993225	0.0224706	140	0.479973	0.0652569
1.625	1.00636	0.0147146	150	0.471674	0.0623355
1.6875	1.0206	0.039587	160	0.464491	0.0678329
1.75	1.0308	0.0626443	165	0.461521	0.0694929
1.875	1.03536	0.0595288	175	0.455044	0.0702457
2	1.03757	0.140597	180	0.454354	0.0690091
2.125	1.05661	0.0580031	185	0.450706	0.0807745
2.25	1.07951	0.047584	190	0.446442	0.0477704
2.5	1.11612	0.0134392	195	0.445182	0.0765487
2.75	1.15096	0.0417291	200	0.443174	0.075495
3	1.18637	0.0549517	212.5	0.436772	0.0750927
3.25	1.2192	0.0958209	225	0.432475	0.0477424
3.5	1.25362	0.034796	237.5	0.429575	0.0770921
3.875	1.27999	0.0602744	250	0.428672	0.0863672
4.25	1.3268	0.0283756	275	0.419821	0.0642036
			300	0.414074	0.0833777
**OXYGEN**					
**E (MeV/n)**	***α* (Gy^−1^)**	***β* (Gy^−2^)**	**E (MeV/n)**	***α* (Gy^−1^)**	***β* (Gy^−2^)**
0.1	0.413736	0.0142512	6	1.09991	0.0179879
0.2	0.389411	0.0289218	7	1.17086	0.0553748
0.25	0.390787	0.0132718	8	1.23791	0.03446
0.3	0.408917	0.0151436	10	1.36301	0.0780824
0.35	0.432501	0.0102363	13	1.53735	0.0380272
0.4	0.457564	0.0140088	15	1.63487	0.0208637
0.45	0.487057	0.0416276	20	1.77359	0.0114724
0.5	0.512859	0.029806	25	1.78011	0.0369413
0.55	0.497537	0.0513708	30	1.69153	0.0243579
0.6	0.519719	0.023812	40	1.43087	0.0687465
0.7	0.561975	0.0147102	50	1.20946	0.0680245
0.75	0.585803	0.0239479	60	1.044	0.0593522
0.8	0.608447	0.00467075	70	0.93624	0.0608797
0.85	0.618215	0.0265444	80	0.854476	0.0671706
0.9	0.636862	0.0320603	90	0.803408	0.0549333
0.95	0.65226	0.0291653	100	0.758682	0.0548792
1	0.670432	0.0378584	110	0.720772	0.0656161
1.25	0.710918	0.0462263	120	0.689713	0.0541748
1.5	0.7642	0.0888616	130	0.659153	0.0666365
1.75	0.794974	0.0780676	140	0.641074	0.0580988
2.25	0.836185	0.0696362	150	0.621408	0.0605031
2.5	0.864835	0.0403956	160	0.602909	0.0606604
2.75	0.880924	0.0148689	180	0.576667	0.0703917
3	0.894613	0.00887251	200	0.556955	0.0680523
3.5	0.941321	0.0402146	250	0.517284	0.0828523
5	1.04036	0.0320689	300	0.493476	0.0861731
			350	0.47441	0.0914356

## Appendix B

**Table A2:** Predictions of *α* and *β* Values for the HSG Cell Line in Response to Hydrogen, Helium, Carbon and Oxygen Ions for the mMKM Model.

**HYDROGEN**					
**E (MeV/n)**	***α* (Gy^−1^)**	***β* (Gy^−2^)**	**E (MeV/n)**	***α* (Gy^−1^)**	***β* (Gy^−2^)**
0.1	1.76015784	0.0615	4.25	0.41269852	0.0615
0.125	1.90316891	0.0615	4.625	0.39792158	0.0615
0.15	1.85167715	0.0615	5	0.37526441	0.0615
0.175	1.79620928	0.0615	6	0.3465522	0.0615
0.2	1.72047753	0.0615	7	0.32547431	0.0615
0.225	1.686197	0.0615	7.5	0.31677023	0.0615
0.25	1.63496363	0.0615	8	0.31103573	0.0615
0.275	1.55673265	0.0615	9	0.30400634	0.0615
0.3	1.50465213	0.0615	10	0.29759482	0.0615
0.325	1.45851417	0.0615	13	0.27563387	0.0615
0.35	1.40890868	0.0615	14	0.25797535	0.0615
0.375	1.37158627	0.0615	14.5	0.26592402	0.0615
0.4	1.34708463	0.0615	15	0.2607293	0.0615
0.425	1.28856167	0.0615	16	0.25896092	0.0615
0.45	1.27640762	0.0615	17	0.2597976	0.0615
0.475	1.22548674	0.0615	18.5	0.24932084	0.0615
0.5	1.20290671	0.0615	20	0.23703403	0.0615
0.525	1.16652015	0.0615	22.5	0.23584525	0.0615
0.55	1.14828801	0.0615	25	0.24512923	0.0615
0.6	1.10455273	0.0615	30	0.23253512	0.0615
0.625	1.0710152	0.0615	35	0.22953456	0.0615
0.65	1.0442441	0.0615	40	0.22510101	0.0615
0.675	1.02892927	0.0615	42.5	0.21525845	0.0615
0.7	1.01230563	0.0615	45	0.21364756	0.0615
0.725	1.00993717	0.0615	50	0.20728247	0.0615
0.75	0.99169901	0.0615	60	0.21160957	0.0615
0.775	0.9504684	0.0615	70	0.21542632	0.0615
0.8	0.93575076	0.0615	72.5	0.21168926	0.0615
0.825	0.93725789	0.0615	75	0.21454225	0.0615
0.85	0.91940491	0.0615	80	0.20557802	0.0615
0.875	0.90710971	0.0615	85	0.20915264	0.0615
0.9	0.88489348	0.0615	87.5	0.21005575	0.0615
0.925	0.85892201	0.0615	90	0.19931656	0.0615
0.95	0.84808765	0.0615	100	0.20679814	0.0615
0.975	0.82971714	0.0615	110	0.20829383	0.0615
0.988	0.85598572	0.0615	115	0.20360297	0.0615
1	0.9810095	0.0615	120	0.20592231	0.0615
1.375	0.81205418	0.0615	125	0.20449326	0.0615
1.438	0.81442601	0.0615	130	0.20444967	0.0615
1.5	0.75449721	0.0615	132.5	0.20764789	0.0615
1.562	0.75771038	0.0615	135	0.20557136	0.0615
1.625	0.73026718	0.0615	140	0.20865446	0.0615
1.688	0.69581744	0.0615	160	0.2021342	0.0615
1.75	0.69183551	0.0615	165	0.19910029	0.0615
1.875	0.66042851	0.0615	170	0.20163043	0.0615
2	0.63047876	0.0615	175	0.20342183	0.0615
2.125	0.61644836	0.0615	180	0.19935637	0.0615
2.25	0.57953347	0.0615	190	0.19497002	0.0615
2.375	0.58510633	0.0615	200	0.19964379	0.0615
2.5	0.54312614	0.0615	212.5	0.19807176	0.0615
2.75	0.52668644	0.0615	225	0.1980282	0.0615
3	0.5225944	0.0615	237.5	0.19863153	0.0615
3.25	0.47132339	0.0615	250	0.20256654	0.0615
3.5	0.45446625	0.0615	275	0.19835696	0.0615
3.875	0.42329655	0.0615	300	0.19917811	0.0615
**HELIUM**					
**E (MeV/n)**	***α* (Gy^−1^)**	***β* (Gy^−2^)**	**E (MeV/n)**	***α* (Gy^−1^)**	***β* (Gy^−2^)**
0.1	1.39368254	0.0615	4.625	0.96230827	0.0615
0.115	1.36937733	0.0615	4.8	0.94225563	0.0615
0.125	1.36745112	0.0615	5	0.88911444	0.0615
0.135	1.36213099	0.0615	5.5	0.83434223	0.0615
0.15	1.38439522	0.0615	6	0.82117505	0.0615
0.175	1.42048374	0.0615	7	0.72771876	0.0615
0.2	1.47155149	0.0615	7.5	0.67973888	0.0615
0.225	1.51566938	0.0615	8	0.65120626	0.0615
0.25	1.45535665	0.0615	9	0.60447989	0.0615
0.275	1.51257501	0.0615	9.5	0.59490674	0.0615
0.3	1.56460825	0.0615	12	0.517091	0.0615
0.325	1.60506986	0.0615	13	0.49108525	0.0615
0.35	1.66200317	0.0615	14	0.48217333	0.0615
0.375	1.70567852	0.0615	14.5	0.45355589	0.0615
0.4	1.75101482	0.0615	15	0.4603493	0.0615
0.425	1.78517233	0.0615	16	0.43637705	0.0615
0.45	1.82903515	0.0615	17	0.42769368	0.0615
0.475	1.86894901	0.0615	18.5	0.40850354	0.0615
0.5	1.89593537	0.0615	20	0.38354342	0.0615
0.525	1.91584789	0.0615	22.5	0.39052976	0.0615
0.55	1.9416978	0.0615	25	0.34625961	0.0615
0.56	1.95247026	0.0615	30	0.34060395	0.0615
0.575	1.96519554	0.0615	35	0.31125112	0.0615
0.58	1.97270031	0.0615	37.5	0.31120437	0.0615
0.6	1.90338008	0.0615	40	0.30110398	0.0615
0.625	1.93381438	0.0615	42.5	0.29806927	0.0615
0.65	1.94856927	0.0615	45	0.28731212	0.0615
0.675	1.96447665	0.0615	50	0.28434712	0.0615
0.7	1.99257377	0.0615	60	0.27235922	0.0615
0.725	2.004059	0.0615	65	0.27475892	0.0615
0.75	2.0092631	0.0615	70	0.25924287	0.0615
0.775	2.02168365	0.0615	72.5	0.25075129	0.0615
0.8	2.03352762	0.0615	75	0.25857623	0.0615
0.825	2.03364266	0.0615	80	0.25831101	0.0615
0.85	2.04461617	0.0615	85	0.2523552	0.0615
0.875	2.04794839	0.0615	87.5	0.23523516	0.0615
0.9	2.04944316	0.0615	90	0.24796673	0.0615
0.925	2.05383802	0.0615	100	0.24530574	0.0615
0.95	2.05227089	0.0615	110	0.23886659	0.0615
0.975	2.05360573	0.0615	120	0.23670685	0.0615
1	2.04932288	0.0615	130	0.23381593	0.0615
1.125	2.03431604	0.0615	132.5	0.23330326	0.0615
1.25	2.00341284	0.0615	135	0.22587891	0.0615
1.312	1.97632077	0.0615	140	0.22961596	0.0615
1.375	1.94453497	0.0615	160	0.22902871	0.0615
1.438	1.93463072	0.0615	165	0.23033065	0.0615
1.5	1.91692309	0.0615	170	0.22880151	0.0615
1.562	1.88227096	0.0615	175	0.22281551	0.0615
1.625	1.82822213	0.0615	180	0.22562653	0.0615
1.688	1.81990989	0.0615	185	0.2232301	0.0615
1.75	1.79166858	0.0615	190	0.21677319	0.0615
1.8	1.76094575	0.0615	195	0.22151075	0.0615
1.875	1.75294194	0.0615	200	0.22526867	0.0615
1.9	1.69542062	0.0615	212.5	0.2183769	0.0615
2	1.67302649	0.0615	225	0.22302907	0.0615
2.125	1.60603852	0.0615	237.5	0.2219778	0.0615
2.25	1.57159	0.0615	250	0.21148815	0.0615
2.375	1.5227767	0.0615	275	0.22256361	0.0615
2.5	1.49062651	0.0615	300	0.2120016	0.0615
2.75	1.37790739	0.0615	400	0.21014623	0.0615
3	1.29101377	0.0615	500	0.21069392	0.0615
3.25	1.23449549	0.0615	600	0.20814372	0.0615
3.5	1.16275574	0.0615	700	0.20445455	0.0615
3.75	1.11019208	0.0615	800	0.20849856	0.0615
3.875	1.07576636	0.0615	900	0.20332779	0.0615
4.25	1.00479394	0.0615	1000	0.20570698	0.0615
**CARBON**					
**E (MeV/n)**	***α* (Gy^−1^)**	***β* (Gy^−2^)**	**E (MeV/n)**	***α* (Gy^−1^)**	***β* (Gy^−2^)**
0.15	0.50339808	0.0615	5	1.55653422	0.0615
0.175	0.48454942	0.0615	6	1.75053464	0.0615
0.2	0.47487403	0.0615	7	1.90877459	0.0615
0.225	0.47187832	0.0615	7.5	1.95249266	0.0615
0.25	0.47407494	0.0615	8	1.99679369	0.0615
0.275	0.45992261	0.0615	9	2.03862693	0.0615
0.3	0.46235642	0.0615	10	2.05601166	0.0615
0.35	0.47192496	0.0615	13	1.97305347	0.0615
0.375	0.47813642	0.0615	14	1.94262411	0.0615
0.4	0.48505401	0.0615	14.5	1.90610348	0.0615
0.45	0.49879305	0.0615	15	1.89554533	0.0615
0.475	0.50483791	0.0615	16	1.85562239	0.0615
0.5	0.51322218	0.0615	17	1.77657221	0.0615
0.525	0.49146793	0.0615	18.5	1.73405714	0.0615
0.53	0.49445111	0.0615	20	1.66676365	0.0615
0.55	0.49573957	0.0615	22.5	1.54318579	0.0615
0.575	0.50445866	0.0615	25	1.46078637	0.0615
0.6	0.51250679	0.0615	27	1.40127589	0.0615
0.625	0.51767073	0.0615	30	1.32642616	0.0615
0.65	0.5256997	0.0615	32	1.27113082	0.0615
0.675	0.53160257	0.0615	35	1.1848265	0.0615
0.7	0.53863325	0.0615	37.5	1.14827529	0.0615
0.725	0.54617082	0.0615	40	1.09587716	0.0615
0.75	0.54341952	0.0615	42.5	1.03951646	0.0615
0.775	0.54739658	0.0615	45	1.01144134	0.0615
0.8	0.55725082	0.0615	50	0.95704141	0.0615
0.825	0.56183319	0.0615	60	0.84947386	0.0615
0.85	0.56804995	0.0615	65	0.79309164	0.0615
0.875	0.57653972	0.0615	70	0.76869037	0.0615
0.9	0.58180655	0.0615	72.5	0.74023123	0.0615
0.925	0.58825069	0.0615	75	0.7394691	0.0615
0.95	0.59650123	0.0615	80	0.7154438	0.0615
0.975	0.60167595	0.0615	85	0.67492106	0.0615
0.988	0.60524041	0.0615	87.5	0.6745586	0.0615
1	0.6126607	0.0615	90	0.66200055	0.0615
1.125	0.64546276	0.0615	100	0.62537666	0.0615
1.25	0.64506518	0.0615	110	0.51090028	0.0615
1.312	0.66343661	0.0615	120	0.56221711	0.0615
1.375	0.6801614	0.0615	130	0.54273609	0.0615
1.438	0.69527217	0.0615	132.5	0.53486851	0.0615
1.5	0.71570436	0.0615	135	0.53097809	0.0615
1.562	0.7343878	0.0615	140	0.52415145	0.0615
1.625	0.74580065	0.0615	150	0.50843461	0.0615
1.688	0.76131236	0.0615	160	0.49663483	0.0615
1.75	0.78269442	0.0615	165	0.48895603	0.0615
1.875	0.80061062	0.0615	175	0.47546677	0.0615
2	0.83450504	0.0615	180	0.47380211	0.0615
2.125	0.8360636	0.0615	185	0.46439192	0.0615
2.25	0.87478362	0.0615	190	0.4597621	0.0615
2.5	0.93848816	0.0615	195	0.45408332	0.0615
2.75	1.0071553	0.0615	200	0.45227578	0.0615
3	1.08229543	0.0615	212.5	0.44305794	0.0615
3.25	1.15264005	0.0615	225	0.43045946	0.0615
3.5	1.21718809	0.0615	237.5	0.42182226	0.0615
3.875	1.28441299	0.0615	250	0.40828412	0.0615
4.25	1.36850829	0.0615	275	0.40622332	0.0615
4.625	1.48784096	0.0615	300	0.39136842	0.0615
**OXYGEN**					
**E (MeV/n)**	***α* (Gy^−1^)**	***β* (Gy^−2^)**	**E (MeV/n)**	***α* (Gy^−1^)**	***β* (Gy^−2^)**
0.1	0.57683641	0.0615	40	1.61813083	0.0615
0.2	0.39461372	0.0615	50	1.41288483	0.0615
0.25	0.38549096	0.0615	60	1.2769869	0.0615
0.3	0.38420183	0.0615	70	1.1465992	0.0615
0.35	0.38874355	0.0615	80	1.0769192	0.0615
0.4	0.39431564	0.0615	90	0.99183267	0.0615
0.45	0.40153681	0.0615	100	0.93563608	0.0615
0.5	0.41036818	0.0615	110	0.90126161	0.0615
0.55	0.39382537	0.0615	120	0.83897904	0.0615
0.6	0.40244051	0.0615	130	0.80282571	0.0615
0.7	0.41948742	0.0615	140	0.76208427	0.0615
0.75	0.42708322	0.0615	150	0.73875254	0.0615
0.8	0.43581879	0.0615	160	0.71921426	0.0615
0.85	0.44460969	0.0615	180	0.67685998	0.0615
0.9	0.45262565	0.0615	200	0.63836255	0.0615
0.95	0.46079407	0.0615	250	0.58416082	0.0615
1	0.47074632	0.0615	300	0.53276501	0.0615
1.25	0.5008777	0.0615	350	0.50905649	0.0615
1.5	0.53970912	0.0615	400	0.48671036	0.0615
1.75	0.57550625	0.0615	0.75	0.36571457	0.0615
2.25	0.63054616	0.0615	1.5	0.43244717	0.0615
2.5	0.662315	0.0615	1.7	0.45753939	0.0615
2.75	0.69868629	0.0615	2.8	0.55556211	0.0615
3	0.74023048	0.0615	5	0.73531098	0.0615
3.5	0.82502643	0.0615	7	0.91601311	0.0615
5	1.02532216	0.0615	8	1.01432123	0.0615
6	1.17652843	0.0615	10	1.18638951	0.0615
7	1.2918773	0.0615	13	1.44146401	0.0615
8	1.42043736	0.0615	15	1.59418043	0.0615
10	1.66855287	0.0615	80	1.45976032	0.0615
13	1.92005086	0.0615	85	1.40898716	0.0615
15	2.00085691	0.0615	90	1.35830835	0.0615
20	2.04557772	0.0615	95	1.32300946	0.0615
25	1.96074999	0.0615	100	1.29277276	0.0615
30	1.85318684	0.0615			
